# Oral Therapy for the Treatment of Transthyretin-Related Amyloid Cardiomyopathy

**DOI:** 10.3390/ijms232416145

**Published:** 2022-12-18

**Authors:** Mario Nuvolone, Maria Girelli, Giampaolo Merlini

**Affiliations:** 1 Department of Molecular Medicine, University of Pavia, 27100 Pavia, Italy; 2 Amyloidosis Research and Treatment Center, Fondazione IRCCS Policlinico San Matteo, 27100 Pavia, Italy

**Keywords:** amyloidosis, cardiac amyloidosis, amyloid cardiomyopathy, transthyretin, ATTR amyloidosis, emerging therapies, oral therapies

## Abstract

The care of systemic amyloidosis has improved dramatically due to improved awareness, accurate diagnostic tools, the development of powerful prognostic and companion biomarkers, and a continuous flow of innovative drugs, which translated into the blooming of phase 2/3 interventional studies for light chain (AL) and transthyretin (ATTR) amyloidosis. The unprecedented availability of effective drugs ignited great interest across various medical specialties, particularly among cardiologists who are now recognizing cardiac amyloidosis at an extraordinary pace. In all amyloidosis referral centers, we are observing a substantial increase in the prevalence of wild-type transthyretin (ATTRwt) cardiomyopathy, which is now becoming the most common form of cardiac amyloidosis. This review focuses on the oral drugs that have been recently introduced for the treatment of ATTR cardiac amyloidosis, for their ease of use in the clinic. They include both old repurposed drugs or fit-for-purpose designed compounds which bind and stabilize the TTR tetramer, thus reducing the formation of new amyloid fibrils, such as tafamidis, diflunisal, and acoramidis, as well as fibril disruptors which have the potential to promote the clearance of amyloid deposits, such as doxycycline. The development of novel therapies is based on the advances in the understanding of the molecular events underlying amyloid cardiomyopathy.

## 1. Introduction

In recent years, the management of systemic amyloidosis has greatly improved due to increased disease awareness, the introduction of accurate diagnostic tools, the development of powerful prognostic and companion biomarkers, and a continuous flow of innovative therapies, which has resulted into the blooming of phase 2/3 interventional studies for both light chain (AL) and transthyretin (ATTR) amyloidosis [1,2,3]. The availability of effective therapies has raised great interest across various medical specialties, particularly among cardiologists who are now identifying cardiac amyloidosis at an unprecedented pace. In all amyloidosis referral centers, we are witnessing a substantial increase in the prevalence of wild-type transthyretin (ATTRwt) cardiomyopathy, which is now becoming the most frequent form of cardiac amyloidosis [4]. The accurate prevalence of ATTRwt among the population is presently being investigated. From studies performed on selected populations, an estimated prevalence for the European standard population ≥75 years was 4.15% in males, 1.03% in females, and 2.59% in the general population, as assessed by myocardial uptake in bone scans [5]. Novel, effective, oral drugs have been recently introduced for ATTR cardiac amyloidosis, which are the focus of this review. The development of novel drugs is based on the advances in the understanding of the molecular events underlying amyloid cardiomyopathy [6].

## 2. Transthyretin Structure and Function

Transthyretin (TTR), also known as prealbumin due to its electrophoretic mobility, is a plasma protein of ≈55 kDa involved in the transport of retinol (vitamin A) and thyroxin (T_4_) [7]. Within the circulation, it has a half-life of 1 to 2 days. Transthyretin plasma concentration ranges between 0.2 and 0.4 g/L (≈5 μM) under physiologic conditions, with decreased levels in the presence of acute phase response and malnutrition [8,9,10].

Plasma TTR is mainly produced by hepatocytes. Additional sites of TTR synthesis include the choroid plexus and the retinal pigment epithelium, contributing to the presence of this protein within the cerebrospinal fluid and the vitreous humour, respectively.

Transthyretin is a homotetrameric protein, formed by the non-covalent association of four identical monomeric subunits of 127 amino acid residues each [11]. Each TTR monomer has a prominent β-sheet secondary structure [12]. The spatial orientation of the four TTR monomers forms two distinct dimer–dimer interfaces, the least stable of which generates two identical T_4_ binding sites [13]. 

The established function of plasma TTR is to transport retinol (in complex with retinol-binding protein, RBP), with approximately ≈0.5 equivalent of Holo-RBP per TTR tetramer [14]. Conversely, more than 99% of T_4_ binding sites of circulating TTR tetramers are unoccupied, as the vast majority of bound plasma T_4_ is carried by albumin or thyroid binding globulin (TBG), which outcompete TTR for T_4_ binding thanks to a higher plasma concentration (albumin) or a higher affinity for T_4_ (TBG) [7,15,16]. Within the cerebrospinal fluid, TTR represents about 25% of total protein content [17] and is the main transporter of T_4_, yet TTR T_4_ binding sites are largely unoccupied also in this case due to the low concentration of T_4_ within this compartment [18].

Beyond its activity as a carrier protein, transthyretin has been implicated in several neuroprotective functions [7,19,20]. In particular, TTR was shown to bind to and sequester Aβ oligomers, thus preventing Aβ aggregation and cytotoxicity, both in in vitro and in vivo models of Alzheimer’s disease [21,22,23,24,25,26,27,28].

## 3. Molecular Mechanisms of ATTR Amyloidosis

Protein folding is a complex process involving thousands of molecules and is highly error-prone. The maintenance of proteome homeostasis, proteostasis, depends on complex quality control mechanisms, mainly intracellular but also extracellular, to promote efficient protein folding and trafficking [29]. The progressive decline of the efficacy of these regulatory processes upon aging [30] is likely to contribute to the increased susceptibility of the elderly population to age-associated protein misfolding diseases. Furthermore, the complex quality control mechanisms can be stressed, and, with aging, eventually overwhelmed, by mutations that destabilize the native conformation, triggering protein aggregation. In systemic amyloidosis, the misfolded proteins aggregate into highly ordered cross-β structured amyloid fibrils accumulating in the extracellular space.

Transthyretin holds a certain degree of intrinsic amyloidogenic propensity, which can become evident upon aging, possibly in relation to the above-mentioned reduction in proteostatic capacity. Indeed, in its wild-type form, TTR can lead to aging-associated amyloid deposition mainly affecting the heart, causing ATTRwt amyloidosis (formerly known as senile cardiac amyloidosis, SCA, or senile systemic amyloidosis, SSA). The observation that myocardial amyloid deposits made of wild-type TTR can be found in about 25% of ultra-octogenarian subjects at autopsy further corroborates the intrinsic amyloidogenicity of TTR [31,32].

At the molecular level, the first and rate-limiting step in the process of TTR amyloid formation is believed to be TTR tetramer dissociation. This is followed by partial denaturation of the native monomer (misfolding) and subsequent aggregation, leading to the formation of oligomers and amyloid fibrils (among other structurally heterogeneous aggregates) [32]. Recently, the combination of proteolytic cleavage and shear forces was shown to promote TTR fibril formation in vitro [33]. Interestingly, ATTR fibrils formed through this mechano-enzymatic-driven amyloidogenic pathway have a morphology and thermodynamic stability, which are closer to ex vivo fibrils compared to ATTR fibrils generated in vitro under acidic, partially denaturing conditions [34]. 

The limited intrinsic amyloidogenic potential of TTR can be significantly exacerbated by one of an ever-growing list of DNA mutations (more than 130 pathogenic mutations in the *TTR* gene reported, www.amyloidosismutation.org). Each mutation results in an amino acid replacement in the TTR polypeptide chain and the production of variant TTR monomers. In heterozygous individuals, variant TTR monomers are incorporated within tetramers in a statistical fashion, leading to the formation of both the wild-type and variant homotetramers, as well as heterotetramers with one, two, or three variant monomers and three, two, or one wild-type monomers, respectively [35].

Destabilizing *TTR* mutations either decrease the quaternary structural stability of TTR tetramers, thus favoring tetramer dissociation (such as the Val122Ile mutation, p.Val142Ile [36,37]), or destabilize TTR monomers, thus promoting misfolding and subsequent aggregation (such as the Leu55Pro mutation, p.Leu75Pro) [36], or both [38]. 

The presence of a destabilizing mutation in the *TTR* gene can lead to the development of a genetic disease termed variant ATTR amyloidosis (ATTRv amyloidosis), mainly affecting the peripheral and autonomous nervous system, the heart, or both. The disorder has an autosomal dominant pattern of inheritance, which is compatible with the toxic-gain-of-function mechanism of the disease described above, and an incomplete penetrance. Allelic frequencies of pathogenic *TTR* mutations are quite variable. Few mutations are rather frequent in selected ethnic groups or geographic areas, such as the Val122Ile mutation, which is carried in 3–4% of African Americans [39,40], and the Val30Met (p.Val50Met) mutation, which is endemic in some areas of northern Portugal [41], northern Sweden [42], Japan [43] in Balearic Islands [44]. 

The clinical onset of ATTRv is variable among different mutations, and sometimes among families or different family members with a given mutation. Overall, ATTRv amyloidosis begins several decades before the age of onset of ATTRwt amyloidosis, thus showing the dramatic acceleration of the amyloidogenic process in vivo because of the presence of the disease-causing destabilizing mutation. In heterozygous individuals affected by ATTRv amyloidosis, both variant and wild-type TTR are found within amyloid deposits. This is further witnessed by the progression of cardiac amyloidosis despite the almost complete removal of variant TTR from the circulation in ATTRv patients undergoing liver transplantation—a sort of surgical gene therapy that has represented the only therapeutic option for selected patients with ATTRv for decades—which is explained by the continuous deposition of wild-type TTR in the myocardium [45,46].

## 4. TTR-Related Cardiac Amyloidosis

In TTR-related cardiac amyloidosis, both prefibrillar, monomeric, and oligomeric species, exert direct proteotoxicity [47], and space-occupying amyloid fibrils, resulting in cardiac stiffness [48], cause heart dysfunction.

Wild-type ATTR amyloidosis manifests with heart failure—more commonly with preserved ejection fraction—arrhythmias, and conduction system disease. For as-of-yet poorly defined mechanisms, ATTRwt amyloidosis has a strong sex and racial preference. Indeed, 80–90% of patients are elderly men, mainly Caucasians, mostly in their 8th or 9th decade of life, when amyloid cardiomyopathy becomes symptomatic and the disease is diagnosed [49,50,51,52,53]. Beyond the heart, wild-type transthyretin can form amyloid deposits in soft tissues, possibly leading to carpal tunnel syndrome, most often bilateral, lumbar spinal stenosis, and spontaneous rupture of the biceps tendon [50,54,55]. In addition, other orthopedic manifestations, including total knee or hip arthroplasty, are more often part of the past medical history among patients with ATTRwt amyloid compared to an age- and sex-matched control population [56]. Of note, these extra-cardiac manifestations typically precede the onset of cardiac symptoms by 5–10 years, suggesting that the process of amyloid formation in vivo is slow and begins several years before leading to symptomatic cardiomyopathy. Myopathy due to interstitial amyloid deposition can be a rare presentation of ATTRwt amyloidosis and precede the onset of cardiomyopathy [57]. Recently, a higher prevalence of neuropathic symptoms in the absence of an alternative diagnosis was described among patients with ATTRwt amyloidosis compared to aged-matched populations [58]. Whether this reflects amyloid deposition within peripheral nerves is presently unknown. 

Variant ATTR amyloidosis can affect almost exclusively the peripheral and autonomous nervous system (formerly termed familial amyloid polyneuropathy, FAP), presenting with length-dependent sensory-motor polyneuropathy and dysautonomia, with heart involvement, eventually occurring only in late disease stages. Alternatively, heart involvement can represent the main manifestation of the disease (formerly familial amyloid cardiomyopathy, FAC), in the presence or absence of polyneuropathy [59]. Organ involvement, age of onset, and clinical presentation are dictated by the underlying mutation and, for the Val30Met mutation, by geography (i.e., the occurrence in endemic versus non-endemic areas). Few mutations can lead to ocular or leptomeningeal amyloidosis [60,61]. Amyloid fibrils from patients with ATTRv amyloidosis consist more commonly of a mixture of intact and fragmented ATTR (type A fibrils), while patients with early onset ATTRv amyloidosis with polyneuropathy from endemic areas have amyloid fibrils consisting of mainly intact ATTR (type B) [62].

At least 94 different mutations in the *TTR* gene have been associated with the development of ATTRv amyloidosis with cardiomyopathy. Males represent about 70% of patients [63,64]. Clinical manifestations include heart failure, arrhythmias, and conduction system disease. Indeed, from a clinical point of view, ATTRv cardiomyopathy can mimic ATTRwt amyloidosis, including for the cardiac uptake of bisphosphonate radiotracers during bone scintigraphy, and genetic testing is required to distinguish between the two disease entities.

Serum levels of the N-terminal natriuretic peptide type B (NT-proBNP), a sensitive biomarker of cardiac dysfunction, with either serum cardiac troponin T or the estimated glomerular filtration rate (eGFR), can stratify patients into different disease stages with different risk categories [51,65]. 

In the recent era of biopsy-free diagnosis of ATTRwt amyloidosis based on the combination of bone scintigraphy, M protein studies (to exclude the possibility of an immunoglobulin light chain amyloidosis, AL), and genetic testing (to exclude hereditary amyloidosis) [66]. ATTRwt cardiomyopathy is diagnosed at earlier stages than before [63]. Such earlier diagnosis translates into better outcomes compared with previous case series, with a median survival of about 5 years from diagnosis [63]. 

## 5. Principles of Therapy

The first disease-modifying therapy against ATTR amyloidosis was liver transplantation, to abolish the hepatic release of the variant TTR within the circulation, and is best employed to halt amyloid polyneuropathy in patients with early onset ATTRv caused by the Val30Met mutation in endemic areas [67].

**Figure 1:**
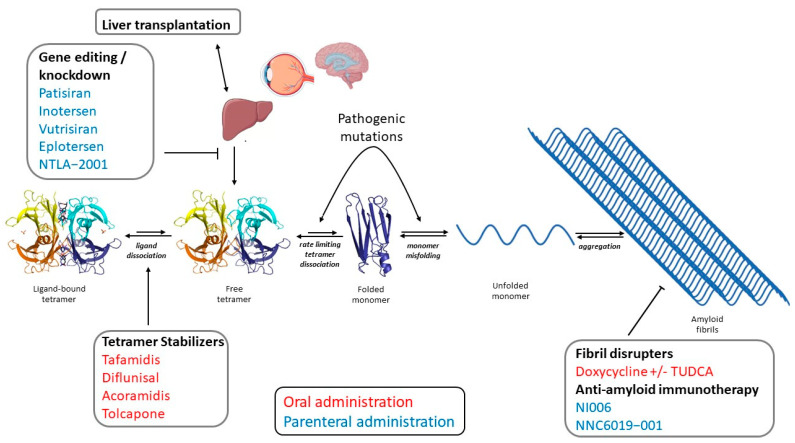
Current and emerging therapies for the treatment of ATTR amyloidosis. Transthyretin, which is mainly synthesized by liver, the retinal pigment epithelium, and the choroid plexus, circulate in body fluids as a homotetramer (crystallographic structure of PDB 2ROX). The rate-limiting step for TTR amyloid formation is TTR tetramer dissociation (unequal arrows denote that the thermodynamic equilibrium is shifted towards TTR tetramers rather than monomeric units), followed by partial denaturation of TTR monomer, leading to aggregation and amyloid formation. Ligands (including the physiologic ligand T_4_) stabilize TTR tetramers. Pathogenic mutations associated with the development of hereditary ATTR amyloidosis (ATTRv) can favor tetramer dissociation, monomer misfolding, or both. Current and emerging therapeutic approaches against ATTR amyloidosis include orthotopic liver transplantation, TTR tetramer stabilizers, *TTR* gene editing, *TTR* gene knockdown, and agents aimed at disrupting amyloid fibrils or favoring amyloid clearance. Pharmacologic therapies include drugs administered through the oral (red) or the parenteral route (blue). TUDCA: tauroursodeoxycholic acid.

Beyond liver transplantation, there are several pharmacological strategies under development for the treatment of ATTR amyloidosis (Figure 1). They are based on the clearer comprehension of the molecular mechanisms involved in the amyloid cascade and include: (1) Suppression of hepatic TTR synthesis through genome editing or *TTR* mRNA knockdown/silencing; (2) TTR tetramer stabilization based on small compounds, and (3) TTR amyloid fibril disruption and/or resorption based on antibodies and/or small compounds. 

Oral treatments include drugs employed to stabilize TTR tetramers or to disrupt amyloid fibrils (Table 1). 

## 6. TTR Tetramer Stabilizers 

The rationale for stabilizing TTR tetramers to prevent amyloid formation stemmed from the compelling observation of a strikingly benign clinical evolution of FAP in a patient carrying the highly penetrant Val30Met mutation in the endemic Portuguese area [94]. Genetic analysis showed that the subject and additional family members with mild disease manifestations were compound heterozygous, carrying the pathogenic Val30Met on one *TTR* allele and a second mutation, Thr119Met (p.Thr139Met), on the other allele [95]. Subsequent biochemical and biophysical studies showed that Thr119Met homotetramer has a 25-fold slower dissociation rate compared to wild-type tetramers [96,97,98]. In addition, the progressive incorporation of Thr119Met subunits into otherwise Val30Met-containing tetramers progressively reduced tetramer dissociation and amyloidogenesis in vitro, resulting in interallelic trans-suppression of misfolding and in line with the initial clinical observation [38,96,97].

Studies using the natural TTR-ligand T_4_ and the nonnative ligand 2,4,6-triiodophenol served as a proof-of-concept that pharmacological TTR tetramer stabilization can reduce amyloid formation [99] and paved the way for both screening and structure-based drug design efforts to identify TTR tetramer stabilizers of therapeutic value to treat ATTR amyloidosis [38].

### 6.1. Tafamidis

A rational, structure-guided screening of a small library of substituted benzoxazoles using a fibril-forming assay led to the identification of tafamidis as a potent kinetic stabilizer of TTR tetramers [100].

Determination of a high-resolution structure of TTR after co-crystallization of wild-type TTR with a 5-molar excess of tafamidis showed that tafamidis binds to the T_4_ binding sites of the TTR tetramer, and it engages in a combination of hydrophobic and ionic interactions to bridge adjacent dimers at the weaker dimer–dimer interface [68]. Kinetic studies showed that tafamidis binds to TTR tetramers with high affinity and negative cooperativity (with dissociation constants of 2 nM and 154 nM for the dissociation constants from the first and second T_4_ binding sites, respectively). In vitro, tafamidis inhibited TTR tetramer dissociation both under denaturing and physiologic conditions, and reduced fibril formation at acidic pH in a dose-dependent manner [68]. Of note, the half-maximal effective concentration (EC_50_) for inhibition of in vitro fibril formation was between 2.7 and 3.2 µM, which corresponds to a tafamidis:TTR molar ratio of 0.75 to 0.90 [68]. This is consistent with the notion that the occupancy of only one of the two T_4_ binding sites is sufficient to kinetically stabilize TTR tetramers [101,102]. In addition, binding studies showed that tafamidis preferentially binds to TTR in human plasma, and dose-dependently stabilizes TTR tetramers in plasma from both healthy subjects and patients carrying a broad range of pathogenic TTR variants [68].

These data motivated the clinical development of this drug, initially in the form of tafamidis meglumine [commercial name: VYNDAQEL; chemical name: 2-(3,5-dichlorophenyl)-1,3-benzoxazole-6-carboxylic acid mono (1-deoxy-1-methylamino-D-glucitol); molecular formula: C_14_H_7_Cl_2_NO_3_/C_7_H_17_NO_5_; molecular weight: 503.33 g/mol] prepared as a capsule containing 20 mg of tafamidis meglumine (corresponding to 12.2 mg of tafamidis free acid) for oral administration. 

The therapeutic effect of TTR tetramer stabilization with tafamidis meglumine was first evaluated in the context of a multi-center, international, randomized, double-blind, placebo-controlled clinical trial on early-stage ATTRv polyneuropathy, recruiting 125 patients. Compared to placebo treatment, oral administration of a single daily dose (QD) of tafamidis meglumine 20 mg slowed the progression of neurological impairment and the decline in patients’ quality of life, while not significantly increasing the rate of adverse events over placebo [69]. Of note, an immunoturbidimetric assay demonstrated TTR tetramer stabilization in 98% of tafamidis-treated patients [69]. Based on these results, tafamidis meglumine was approved for the treatment of adults with early-stage symptomatic ATTRv polyneuropathy in more than 40 countries, but not in the United States.

Transthyretin tetramer stabilization was confirmed also upon oral treatment with tafamidis meglumine 20 mg once daily in patients with ATTRv with polyneuropathy and mutations other than Val30Met in 100% of evaluable patients [103]. A subsequent open-label, single-treatment arm study failed to show a clinical benefit in patients suffering from ATTRv with polyneuropathy with the Val30Met mutation and an advanced disease stage at the time of treatment initiation, thus showing the importance of starting pharmacological stabilization of TTR tetramers at earlier disease stages [104]. Of note, based on cumulative data from the Val30Met patients in the 18-month double-blind registration study and its 12-month open-label extension study, the non-Val30Met patients of the 12-month open-label study, and both patient groups in the ongoing 10-year extension study, Kaplan Meier estimates suggest that long-term tafamidis treatment may confer a survival benefit in patients with ATTRv with polyneuropathy [105].

The initial therapeutic success of tafamidis-based TTR tetramer stabilization for the treatment of ATTRv polyneuropathy prompted further clinical development of this drug to treat ATTR cardiomyopathy. 

Considering the involvement of the autonomous nervous system in ATTRv with polyneuropathy and the risk for conduction disorders in ATTR cardiomyopathy, the potential effect of tafamidis on the corrected QT interval was evaluated in the context of a randomized, three-treatment, three-period, six-sequence crossover study with single oral doses of placebo, a positive control (moxifloxacin 400 mg), and tafamidis (400 mg) in 42 healthy volunteers [106]. Tafamidis dosing was chosen to achieve a supra-therapeutic Cmax of ~20 µg/mL. This study demonstrated that a supra-therapeutic single 400 mg oral dose of tafamidis does not prolong the corrected QT interval and is well tolerated in healthy volunteers [106].

A phase 2, multi-center, open-label, single-treatment arm study evaluated the safety and effects on TTR tetramer stability in patients with ATTR cardiomyopathy treated with oral tafamidis meglumine 20 mg once daily over a 12-month period [107]. Thirty-one patients with ATTRwt cardiomyopathy were enrolled (29 males, 94%), with a median age of 77 years. Distribution according to the New York Heart Association (NYHA) classification of heart failure was 16%, 81%, 3%, and 0% for NYHA class I, II, III, and IV, respectively, and 65% of patients had atrial fibrillation. Transthyretin tetramer stabilization was achieved in 97% and 89% of patients at 6 weeks and 12 months, respectively [107]. Overall, adverse events reported during the study were considered in line with expectations for an elderly population with significant heart disease and comorbid conditions. This was taken to indicate that treatment with tafamidis meglumine 20 mg in patients with ATTRwt cardiomyopathy was well tolerated, even though the lack of a parallel control arm limited the ability to conclude on safety and efficacy on outcome measures [107].

The therapeutic effect of tafamidis meglumine was evaluated in the context of the Transthyretin Amyloidosis Cardiomyopathy Clinical Trial (ATTR-ACT). This was a multi-center, international, double-blind, placebo-controlled, phase 3 clinical trial where 441 patients with heart failure due to ATTR cardiomyopathy were randomly assigned in a 2:1:2 ratio to receive 80 mg or 20 mg of tafamidis meglumine, or placebo, orally once daily for 30 months [70]. The median age of enrolled patients was 75 years and 90% of patients were males. Seventy-six percent of patients had ATTRwt cardiomyopathy, while the remaining subjects had ATTRv cardiomyopathy, with Val122Ile, Thr60Ala, and Ile68Leu being the most common pathogenic mutations. Distribution according to the NYHA classification was 8%, 60% and 32%, for NYHA class I, II, and III, respectively, while patients with NYHA class IV heart failure were not eligible. Patients were stratified based on *TTR* genotype and NYHA class. Tafamidis meglumine 80 mg was administered as four oral capsules of tafamidis meglumine 20 mg. Treatment adherence (predefined as taking ≥80% of scheduled doses) was 97% for both tafamidis and placebo. In addition, the incidence and types of adverse events were similar in the two groups and there was no significant difference in the safety of the two doses of tafamidis meglumine. Overall, these data indicate that treatment was well tolerated [70]. Importantly, compared to placebo, treatment with tafamidis meglumine was associated with a statistically significant reduction in all-cause mortality across all subgroups and reduced cardiovascular-related hospitalizations in patients with NYHA I and II, but not in NYNA III, at study enrolment [70]. 

Tafamidis-treated patients who were in NYHA III functional class at the study beginning showed a higher hospitalization rate with respect to the placebo group, presumably due to longer survival during a more severe period of disease [70]. To account for survivor bias, a posthoc analysis on the subset of patients alive at 30 months showed that tafamidis treatment was associated with a lower risk of cardiovascular-related hospitalization also in NYHA III patients [108]. In addition, differences in all-cause mortality and cardiovascular hospitalizations between the treatment groups emerged first at 18 months from treatment initiation. According to model-based analyses, baseline predictors of the outcome included greater 6 min walking distance, higher left ventricular ejection fraction, and lower blood urea nitrogen, and N-terminal pro-B-type natriuretic peptide concentrations [109]. Collectively, these observations underscore the importance of an early diagnosis and rapid initiation of etiologic therapy [110]. Treatment with tafamidis meglumine also slowed the decline in functional capacity and quality of life at 30 months, with differences compared to the placebo group emerging already at 6 months [70,111]. Moreover, while ATTRv patients showed a poorer prognosis compared to ATTRwt in the placebo arm, the reduction in mortality and functional decline with tafamidis treatment was similar in both disease subtypes [112]. Based on the results of the ATTR-ACT trial, tafamidis was approved for the treatment of ATTR cardiomyopathy in Japan, the United States, the United Arab Emirates, Brazil, Canada, and the European Union. 

To enable a single oral capsule for daily administration for patient convenience, a novel formulation containing the free acid tafamidis was developed [commercial name: VYNDAMAX; chemical name: 2-(3,5-dichlorophenyl)-1,3-benzoxazole-6-carboxylic acid; molecular formula: C_14_H_7_Cl_2_NO_3_; molecular weight: 308.12 g/mol]. The tafamidis 61 mg capsule corresponds to an 80 mg tafamidis meglumine dose (4 × 20 mg capsules), with bioequivalence of the two formulations formally proven in the context of a single-center, open-label, randomized, 2-period, 2-sequence, crossover, multiple-dose phase 1 study on 30 healthy volunteers [113].

In the ATTR-ACT trial, median overall survival was not achieved in either treatment arm, with 57% of placebo-treated patients and 71% of tafamidis meglumine-treated patients alive at the end of the 30-month study period. However, a subsequent survival extrapolation analysis estimated a median overall survival for the placebo of 35 months, compared to 53 months for the experimental drug [114]. Patients who completed the ATTR-ACT trial could enroll in a long-term extension study, continuing with the same dose of tafamidis or, if previously in the placebo arm, 2:1 randomized to tafamidis meglumine 80 or 20 mg daily. All patients subsequently transitioned to tafamidis free acid 61 mg per protocol amendment. In this study, patients with continuous tafamidis treatment had a substantially reduced mortality than those first treated with a placebo, stressing once more the importance of early treatment initiation in ATTR cardiomyopathy [115]. In addition, combined data from the ATTR-ACT trial and the long-term extension study demonstrated a greater survival benefit with tafamidis 80 vs. 20 mg, without increased incidence of adverse events between the two dosing groups, supporting tafamidis meglumine 80 mg (or tafamidis free acid 61 mg) as the optimal dose [116].

Data from a prospective multi-center disease registry of unmatched patients with ATTR cardiomyopathy showed reduced mortality in tafamidis-treated patients aged 80 years or more compared with untreated patients, with a more pronounced survival benefit in subjects with early-stage disease [117,118]. 

A clinical benefit of tafamidis treatment, in terms of prolonged major cardiovascular outcome-free survival, lower deterioration of structural and functional changes of the left ventricle, and myocardial work, was also reported outside the frame of clinical trials, in retrospective series of real-life patients [119,120,121,122]. 

### 6.2. Diflunisal

Drug screening efforts led to the initial observation that several non-steroidal anti-inflammatory drugs (NSAIDs) could bind to the T_4_ binding site of TTR tetramers with high affinity [123,124,125]. These included diflunisal, which was shown to bind to TTR tetramer with negative cooperativity (with values of 75 nM and 1500 nM for the dissociation constants from the first and second T_4_ binding sites, respectively) [97]. In addition, diflunisal could inhibit tetramer dissociation and in vitro fibril formation of wild-type TTR, as well as of the most common disease-associated TTR variants (Val30Met, Val122Ile, Thr60Ala, Leu58His, and Ile84Ser) [126,127,128]. 

On the one hand, the limited selectivity of diflunisal for TTR in human plasma prompted the search for diflunisal analogs with anti-fibrillogenic activity but increased TTR selectivity based on structure-guided drug design [129]. On the other hand, the excellent oral bioavailability of diflunisal, and the fact that this was an FDA-approved NSAID motivated testing of the potential therapeutic effect of diflunisal to treat ATTR amyloidosis, could allow the possibility of repurposing diflunisal for this novel, unanticipated indication.

Indeed, diflunisal [commercial name: DOLOBID; chemical name: 2′, 4′-difluoro-4-hydroxy-3-biphenylcarboxylic acid; molecular formula: C_13_H_8_F_2_O_3_; molecular weight: 250.20 g/mol] is a non-steroidal drug with analgesic, anti-inflammatory, and antipyretic properties. It is indicated for acute or long-term use for symptomatic treatment of mild to moderate pain, osteoarthritis, and rheumatoid arthritis. It is available in 250 and 500 mg tablets for oral administration. 

The exact mechanisms of the analgesic and anti-inflammatory actions of diflunisal are not known. Diflunisal is a prostaglandin synthetase inhibitor and it may reduce prostaglandin levels in peripheral tissues. It is, indeed, a peripherally-acting non-narcotic analgesic drug, and does not lead to habituation, tolerance, or addiction.

As with all NSAIDs, diflunisal may cause an increased risk of serious cardiovascular thrombotic events, myocardial infarction, and stroke, with patients with cardiovascular disease or risk factors for cardiovascular diseases being at greater risk. Similarly, as with all NSAIDs, diflunisal may cause an increased risk of serious gastrointestinal adverse events including bleeding, ulceration, and perforation of the gastrointestinal tract, with elderly patients being at higher risk for serious gastrointestinal events. The concomitant use of diflunisal and oral anticoagulants may prolong prothrombin time and increase the risk for serious gastrointestinal bleeding. Although less common than cardiovascular and gastrointestinal risks, NSAIDs can cause kidney toxicity through the inhibition of prostaglandin and thromboxane synthesis, leading to renal vasoconstriction and consequently reduced renal perfusion and aberrant renal function. 

Despite potential concerns stemming from these warnings linked to the NSAID activity of diflunisal, the potential effect of this drug to treat ATTR amyloidosis has been tested first in the context of ATTRv with polyneuropathy and, subsequently, for ATTR cardiomyopathy.

In a phase 1 trial on healthy volunteers, oral administration of diflunisal 250 mg twice daily (BID) led to a diflunisal serum concentration of 146 ± 39 µM after one week, and was sufficient to bind TTR tetramers, slow their dissociation, and reduce in vitro fibrillization, in line with kinetic stabilization of TTR [71].

Subsequently, an investigator-initiated, international, multi-center, randomized, double-blind, placebo-controlled study was performed to determine the effect of diflunisal on polyneuropathy progression in patients with ATTRv with polyneuropathy [72]. The study was conducted on 130 patients randomized 1:1 for diflunisal 250 mg twice daily or placebo for two years, upon stratification based on *TTR* mutation (Val30Met or other) and study site. Patients with NYHA IV heart failure, estimated creatinine clearance <30 mL/min, and ongoing anticoagulation were excluded. Due to attrition, likelihood-based modeling and multiple imputation analysis were performed. The safety profile was similar in the two treatment arms. In terms of efficacy, compared to placebo, oral treatment with diflunisal 250 mg twice daily for 2 years reduced the rate of progression of neurological impairment and preserved the quality of life, suggesting clinical benefit of this treatment for ATTRv with polyneuropathy [72].

Long-term effects of diflunisal treatment in ATTRv with polyneuropathy are reported in an open study conducted on 40 Japanese patients with ATTRv (both Val30Met and non-Val30Met mutations) who were not candidates for liver transplantation [73]. Notably, 85% of patients had cardiomyopathy in association with polyneuropathy. Patients were treated with oral diflunisal 250 mg twice daily (and histamine type-2 receptor antagonist or proton pump inhibitor to prevent gastrointestinal bleeding) and had a median follow-up of 38 months (up to 116 months). Overall, long-term treatment with diflunisal was well tolerated, with two dropouts due to renal failure and one owing to thrombocytopenia. Diflunisal treatment increased TTR levels and stabilized TTR tetramers in all patients. Of note, ulnar compound muscle action potential amplitude, cardiac wall thickness, and ejection fraction were not deteriorated after 24 months of treatment, suggesting sustained clinical effects [73]. 

To assess whether diflunisal could be safely administered to patients with ATTR cardiomyopathy and to gather preliminary data on efficacy, a single-arm, open-label phase 1 study was performed [74]. Patients with active or recent gastrointestinal bleeding and patients with eGFR <30 mL/min/m^2^ were excluded. Thirteen patients with ATTR cardiomyopathy (seven with ATTRwt and six with ATTRv cardiomyopathy) were treated with oral diflunisal 250 mg twice daily (along with histamine receptor antagonist or proton pump inhibitor). The median follow-up time was 321 days. Diflunisal treatment was well tolerated. A median 6% decline ineGFR was noted. One patient discontinued diflunisal treatment due to rapidly developed volume overload. Left ventricular mass, ejection fraction, BNP, and troponin I levels did not significantly change over the course of treatment. These results were taken to suggest that diflunisal can be safely administered to compensated patients with ATTR cardiomyopathy [74]. Another retrospective study reported the outcome of 23 patients with ATTR cardiomyopathy (13 ATTRwt and 10 ATTRv) treated with diflunisal (250 mg twice daily in 20 patients and 500 mg twice daily in three patients with mainly polyneuropathy symptoms). A proton-pump inhibitor was co-administered in eight patients, and seven subjects were on anticoagulant therapy while on diflunisal. The median duration of therapy was 15 months (up to more than 7 years). Diflunisal treatment was well tolerated. Three patients were withdrawn from diflunisal therapy due to gastrointestinal side effects (decreased appetite, epigastric pain, and erosive gastritis in one patient each) and one for dizziness. There was a transient increase of serum creatinine (+0.31 mg/dL) in one case. No clinically significant bleeding event occurred [75]. 

Additional data on diflunisal treatment in patients with ATTR cardiac amyloidosis originate from a few case series on a limited number of patients, which also included untreated patients as a comparator group. In a retrospective study on 123 consecutive patients with ATTRv amyloidosis (of which 76 had heart involvement), 41 patients received diflunisal treatment [76]. Seven patients discontinued diflunisal due to transient renal dysfunction. Of the remaining 34 patients who continued diflunisal treatment, speckle-tracking echocardiography showed improvements in apical left ventricle (LV) rotation and torsion, without deterioration of longitudinal and radial strains, at 1 year, whereas a progressive impairment in longitudinal and basal LV radial function with unchanged global circumferential shortening and torsion were reported in untreated patients from this series [76]. In another study, the clinical course of 81 patients with ATTR cardiomyopathy (53 with ATTRwt and 28 with ATTRv) was retrospectively evaluated [77]. Of these, 33 patients were treated with oral diflunisal 250 mg twice daily, while the remaining served as an untreated control group for comparison. Treated patients were younger and had significantly lower BNP levels at diagnosis, possibly reflecting a higher tendency to start diflunisal treatment in younger patients with lower disease burden. The median follow-up of this study was 1 year, during which diflunisal-treated patients showed higher TTR levels, congruent with diflunisal-based tetramer stabilization, as well as favorable differences in left atrial volume index and cardiac troponin I [77]. A further study reported increased survival of ATTR cardiomyopathy patients treated with TTR stabilizers (*n* = 29, of which *n* = 13 on diflunisal) with respect to untreated subjects (*n* = 91) [78]. In addition, in this study, treated patients had more favorable disease characteristics at baseline. The authors concluded that diflunisal at a dose of 250 mg orally twice a day is generally well tolerated by patients with cardiac ATTR amyloidosis who are not acutely decompensated, have eGFR >45 mL/min/m^2^, and are not on high-dose diuretics, with approximately 10% of such patients still not tolerating the treatment [78]. Overall, while these studies are in agreement with the notion that diflunisal is generally well tolerated in patients with ATTR cardiac amyloidosis, with treatment discontinuation occurring in a minority of cases [130], definitive efficacy data from controlled clinical trials in this clinical setting are still missing.

### 6.3. AG10/Acoramidis

Using high throughput screening coupled with structure-based drug design, the fit-for-purpose TTR tetramer stabilizer AG10 was recently developed [80,81]. Biochemical analyses showed that AG10 binds to TTR with high affinity and negative cooperativity (Kd1 = 4.8 nM and Kd2 = 314 nM). Compared to tafamidis, AG10 was more potent in kinetically stabilizing TTR tetramers from both wild-type and Val1222Ile TTR and at inhibiting amyloid formation in vitro. AG10 also showed superior selectivity for TTR in human serum, and superior stabilization of TTR tetramers from patients with ATTR cardiomyopathy (both ATTRwt, and ATTRv associated with the Val122Ile mutation, either in homo- or heterozygosity), compared to tafamidis. In addition, AG10 protected the cardiomyocyte cell line AC16 from the proteotoxicity of amyloidogenic V122I-TTR in vitro, while not affecting viability and proliferation on four cell lines [81]. Noteworthy, AG10 proved good oral bioavailability, dose-dependent TTR occupancy, and lack of toxicity upon subacute administration in rats and dogs [81,82]. 

Subsequent comparisons of AG10 with other TTR tetramer stabilizers, including tafamidis, diflunisal, and tolcapone, showed that this molecule has superior TTR occupancy and stabilization in serum [82,83]. 

These data supported further clinical development of AG10. The corresponding drug substance of AG10 is the hydrochloride salt of AG10, subsequently named acoramidis [chemical name: 3-(3-(3,5-dimethyl-1H-pyrazol-4-yl)propoxy)-4-fluorobenzoic acid; molecular formula: C_15_H_18_FClN_2_O_3_,; molecular weight: 328.77]. In a phase 1, first-in-human, randomized, double-blind, placebo-controlled study, safety, tolerability, pharmacokinetics, and pharmacodynamics (in terms of ex vivo stabilization) of orally administered acoramidis were evaluated in 32 healthy adult volunteers (8 exposed to placebo and 24 to AG10 in different cohorts) [79]. Acoramidis was administered orally as a single dose (from 50 mg to 800 mg) or as multiple doses (from 100 mg to 800 mg) every 12 h for 12 days. Acoramidis was well tolerated, and there were no safety signals of clinical concern [79]. Pharmacokinetics studies showed a time to maximum concentration of less than 1 h and a half-life of ≈25 h [79]. Pharmacodynamics studies showed a >90% stabilization of TTR at steady state on the highest dose tested, and increased TTR levels after 12 days of acoramidis treatment compared to baseline levels [79]. 

Consistent results in terms of overall good tolerability, similar exposures following sustained oral dosing and TTR stabilization were obtained in the context of a phase 2, randomized, placebo-controlled, dose-ranging study of acoramidis in patients with symptomatic ATTR cardiomyopathy. In this study, 49 patients with ATTR cardiomyopathy (median age 74 years, 92% males, 14 with ATTRv) and NYHA class II or III, were randomized 1:1:1 to acoramidis 400 or 800 mg, or placebo twice daily for 28 days. Of note, baseline serum TTR levels in treated subjects was below normal in 80% and 33% of patients with ATTRv and ATTRwt, respectively, and acoramidis treatment restored serum TTR to the normal range in all subjects [84]. 

An international, multi-center, phase 3, placebo-controlled trial on the safety and efficacy of AG10 800 mg orally twice daily in ATTR cardiomyopathy is currently ongoing (NCT03860935). This trial enrolled 632 patients with ATTR cardiac amyloidosis (both ATTRwt and ATTRv), with NYHA class I-III symptoms. Topline results from month 12 have been recently released and show lack of improvement in change from baseline in 6 min walking distance in the acoramidis arm over the placebo arm [85]. Conversely, acoramidis treatment was associated with increased serum TTR levels, lower increase of NT-proBNP levels and improved patients-reported quality of life compared to placebo [85]. The trial is ongoing and results at month 30 are eagerly awaited.

### 6.4. Tolcapone

A screening of a small library of drugs under clinical development or already in clinical use for their potential to inhibit TTR aggregation in vitro led to discovery of tolcapone as a promising candidate [86]. Tolcapone was shown to bind to the T_4_ pocket of TTR tetramers with higher affinity than tafamidis, to stabilize the dimer–dimer interface of the tetramer, to inhibit TTR fibril formation in vitro, and to exert cytoprotective effects against cytotoxic TTR aggregates in AC16 human cardiomyocyte cells [86]. In addition, oral administration of tolcapone results in T_4_ displacement and TTR tetramer binding in FAP transgenic mice expressing human TTR, in healthy volunteers, in asymptomatic carriers of *TTR* pathogenic mutations, and in patients with ATTR amyloidosis, providing the rationale for the clinical development of tolcapone to treat ATTR amyloidosis [86,87]. 

Tolcapone [commercial name: TASMAR; chemical name: 3,4-dihydroxy-4′-methyl-5 nitrobenzophenone; molecular formula: C_14_H_11_NO_5_; molecular weight: 273.25 g/mol] is an orally active cathecol-O-methyltransferase (COMT) inhibitor and is used in the treatment of Parkinson’s disease as an adjunct to levodopa/carbidopa therapy. Albeit tolcapone enters the central nervous system to a minimal extent, animal studies have shown that this drug can indeed inhibit central COMT activity. 

The ability of tolcapone to cross the blood–brain barrier and its potential to bind to TTR within the cerebrospinal fluid make this drug particularly attractive for repurposing efforts aimed at treating ATTRv amyloidosis with leptomeningeal involvement. Noteworthy, a recent study has demonstrated binding—and kinetic stabilization—of tolcapone to three TTR variants associated with leptomeningeal amyloidosis (Ala25Thr, Val30Gly and Tyr114Cys) [88]. There are at present no trials exploring the potential therapeutic use of tolcapone in ATTR cardiomyopathy.

## 7. Amyloid Fibril Disruptors: Doxycycline

Interest on anthracyclines as potential amyloid fibril disrupters dates back to 1995, based on the clinical observation that 4′-iodo-4′-deoxy-doxorubicin (IDOX) can induce amyloid resorption in patients with AL amyloidosis [131]. Biochemical studies demonstrated that IDOX could bind to all five main types of amyloid fibril tested (AL, AA, ATTR, Aβ and Aβ_2_M) and inhibit amyloid fibril formation in vitro and in vivo [131]. Due to antracycline cardiotoxicity, shared by IDOX, subsequent work focused on the structurally related doxycycline. This molecule could disaggregate and detoxify amyloid fibrils made of the aggressive TTR variant Leu55Pro in vitro [89]. Administration of doxycycline in the drinking water to aged transgenic mice expressing human Val30Met TTR resulted in the complete abrogation of amyloid deposition, but not of pre-fibrillar aggregates, after 3 months of treatment, suggesting that doxycycline can disrupt TTR amyloid fibrils in vivo [90]. In the same animal model, combined cycled oral treatment with doxycycline and the biliary acid tauroursodeoxycholic acid (TUDCA) further reduced TTR deposition, both as pre-fibrillar aggregates and congophilic amyloid deposits, and associated tissue markers, proving the synergistic effect of these two drugs in the range of human tolerable doses, thus prompting clinical development for this combination. 

Doxycycline hyclate [commercial name: DORYX; chemical name: [4S(4aR,5S,5aR,6R,12aS)] 4-(dimethylamino)-1,4,4a,5,5a,6,11,12a-octahydro-3,5,10,12,12a-pentahydroxy-6methyl-1,11-deoxonapthtacene-2-carboxamide monohydrochloride, compound with ethyl alcohol (2:1), monohydrate; molecular formula: C_22_H_24_N_2_O_8_, HCl, ½ C_2_H_6_O, ½ H_2_O; molecular weight: 512.9 g/mol] is a broad-spectrum antibiotic synthetically derived from oxytetracycline, in a delayed-release formulation for oral administration [132]. It is indicated for the treatment of rickettsial infections and anthrax, as well as several sexually transmitted infections, respiratory tract infections, and ophthalmic infections [133]. 

Adverse reactions of tetracyclines include gastrointestinal manifestations, photosensitivity, benign intracranial hypertension, hemolytic anemia, thrombocytopenia, neutropenia, and eosinophilia [133,134]. In addition, tetracyclines may depress plasma prothrombin activity, and patients on anticoagulant therapy may require downward adjustment of their anticoagulant dosage [135,136].

Initial clinical data on ATTR amyloidosis derive from a phase II, open-label study to evaluate the efficacy, tolerability, safety, and pharmacokinetics of oral doxycycline (100 mg twice daily) and TUDCA (250 mg three times/day) administered continuously for 12 months in patients with TTR-related amyloidosis [91]. Twenty patients were enrolled (including 17 patients with ATTRv, 2 patients with ATTRwt, and 1 patient developing ATTR amyloidosis as the result of a domino liver transplant). The pharmacokinetics studies carried out at 6 months from treatment initiation documented therapeutic concentrations of doxycycline, with pre-dose plasma levels of 7 ± 2.4 μg/mL and a mean increase of 1.3 μg/mL, 2 h after the assumption of 100 mg of the drug [91]. No serious adverse events (≥Grade 3) were registered. Treatment discontinuation occurred because of gastric pain in one case and because of persistent nausea and loss of appetite in another case. No clinical progression of cardiac involvement or polyneuropathy was seen during the 12-month study period.

In another phase 2, open-label study, the effect of oral doxycycline (200 mg/day for 4 weeks, with intermittent discontinuation for 2 weeks), and ursodeoxycholic acid (UDCA, 750 mg/day) was evaluated [92]. Ursodeoxycholic acid was employed instead of the related TUDCA, since the latter was not available in the country where the study was performed (Sweden). There were 28 enrolled patients, of which 27 had ATTRv and 1 ATTRwt. Cardiac involvement was present in all patients and peripheral nervous system involvement was present in 26 patients. This study was flawed by an 86% dropout rate, due to voluntary dropouts, side effects, or treatment failure (defined by a >30% increase of NT-proBNP from baseline value), with an increase of NT-proBNP levels or worsening of neurologic function in most of the evaluable patients [92].

The effect of the combination of doxycycline and TUDCA in ATTR cardiomyopathy was retrospectively evaluated in a series of 53 patients followed at one institution. Among these subjects, treatment was not tolerated in six cases (11%), due to photosensitivity or gastrointestinal discomfort. Of the remaining 47 evaluable patients, the median age was 71 years, and 41 (87%) were male. Forty-two patients had ATTRwt and five had ATTRv amyloidosis. Twenty-two patients (47%), were in NYHA class III or IV. One-third of patients were on anticoagulant therapy. The median follow-up was 22 months (up to 30 months), during which there were no signs of clinical, biochemical, or echocardiographic progression for most of the treated patients [93]. A multi-center, randomized phase 3 study on doxycycline and TUDCA plus standard supportive therapy versus standard supportive therapy alone is currently ongoing (NCT03481972).

## 8. Discussion

Recent years have witnessed unprecedented and unanticipated successes in the treatment of ATTR amyloidosis. Concerted efforts of academia, pharmaceutical companies, and patient organizations have explored the full spectrum of drug discovery in its complexity, from high throughput screenings and repurposing of old safe drugs for novel indications, to structure-guided drug design of fit-for-purpose new small molecules, from immunotherapy to pioneering gene editing and silencing approaches [137]. 

Beyond the benefit of employing innovative technologies and approaches for drug discovery, such therapeutic success is to a great extent due to the increased knowledge of the molecular mechanisms of the disease. In particular, therapeutic efforts have been directed against the following three key steps in the pathogenesis of the disease: (1) TTR synthesis; (2) TTR tetramer dissociation leading to misfolding and aggregation of monomers; (3) aggregated species and amyloid fibrils exerting a noxious effect in affected tissues.

While the oral therapies presented here include drugs to stabilize TTR tetramers (tafamidis, diflunisal, acoramidis, tolcapone) or to disrupt amyloid fibrils (doxycycline), recent groundbreaking work has added RNA targeting and gene editing therapies to the armamentarium to fight ATTR amyloidosis [138]. These parenterally administered agents act at the initial step of the pathogenesis and afford *TTR* gene editing/knocking down, leading to the conspicuous reduction of the amyloidogenic precursor. From work on AL amyloidosis, such a strategy has proven to be extremely effective, resulting in the dramatic modification of the natural history of the disease [139]. Indeed, the antisense oligonucleotide (ASO) inotersen and the small interfering RNA (siRNA) patisiran have recently demonstrated therapeutic effect on ATTRv amyloidosis with polyneuropathy in the context of controlled phase 3 trials [140,141]. These agents, together with novel gene silencers, with improved pharmacodynamics, are now under evaluation in controlled trials in patients with ATTR cardiomyopathy [142,143,144,145]. Topline results from the ongoing clinical trial investigating patisiran for ATTR cardiac amyloidosis are positive. The trial met both the primary endpoint (a statistically significant improvement in 6 min walk test) as well as the first secondary endpoint (a statistically significant improvement in patients-reported quality of life) in the patisiran arm compared to the placebo arm, with a favorable safety and tolerability profile of the investigational drug [146]. Even more recently, the safety and pharmacodynamic effects of single escalating doses of NTLA-2001, a lipid nanoparticle encapsulating messenger RNA for Cas9 protein and a single guide RNA targeting the *TTR* gene to accomplish in vivo gene editing, have been investigated in six patients with hereditary ATTR amyloidosis with polyneuropathy, within an ongoing phase 1 clinical study [147]. Noteworthy, administration of NTLA-2001 led to dose-dependent decreases in serum TTR protein concentrations (up to 87%) and was associated with only mild adverse events [147]. Preclinical studies confirmed a durable TTR suppression through *TTR* gene knockout after a single dose, with no evidence of off-target editing [147]. This pioneering work holds the potential to profoundly impact on the natural history of ATTR amyloidosis [148]. Outside the realm of ATTR amyloidosis treatment, this study also provided proof-of-concept evidence for therapeutic, in vivo genome editing [149,150].

In addition to these highly effective therapies targeting the upstream key events of the amyloid cascade, elimination of already formed amyloid fibrils from affected tissues represents a desirable therapeutic goal, possibly facilitating functional recovery of involved organs. However, this remains a largely unmet medical need at present, for ATTR cardiomyopathy and for systemic amyloidoses more in general, as the potential therapeutic effect of doxycycline awaits scrutiny in the context of controlled clinical trials [151] and several recent attempts at promoting amyloid clearance through immunotherapy have been unfruitful [152]. This area of research deserves further investigations.

With the availability of effective etiologic therapies, the medical community has to obtain the best use out of them. Evidence from clinical trials on ATTR cardiomyopathy, as well as work on other systemic amyloidoses, highlight the importance of an early treatment initiation, at an early disease stage, before advanced organ damage has occurred [139]. Early treatment initiation means early diagnosis. For ATTR cardiomyopathy, the increased disease awareness, together with refined imaging techniques and the possibility to diagnose the disease through a judicious combination of bone scintigraphy, M protein studies, and genetic testing, even in the absence of a tissue biopsy, are enabling more and earlier diagnoses, at disease stages more likely to benefit from etiologic therapeutic interventions [63,153]. 

In this context, a special case is represented by pre-symptomatic carriers of *TTR* gene mutations possibly associated with the development of ATTRv cardiomyopathy. As genetic testing, also in the forms of whole exome sequencing or gene panels through next generation sequencing, is becoming more accessible, it is expected that the number of mutation carriers identified will significantly increase in future years. Based on the incomplete penetrance of TTR mutations from one side, and on the availability of effective, safe drugs to treat this condition, the best screening protocol to identify early disease manifestations and the optimal timing and modality of treatment initiation in this clinical setting will have to be defined.

As novel therapies for ATTR cardiomyopathy become available, novel questions and challenges arises. The comparative effects of different agents will have to be established, ideally in the context of controlled clinical trials. Similarly, the potential synergistic effect of pathogenetically meaningful combinations of drugs (i.e., a gene silencer or tetramer stabilizer with an amyloid disruptor to inhibit de novo amyloid formation and eliminate already formed amyloid; possibly, a gene silencer with a tetramer stabilizer to stabilize residually synthetized TTR) will need to be assessed. In addition, pharmacoeconomic aspects will have to be faced. Extremely high costs of innovative drugs may limit access to effective therapies and represent an increasing economic burden to patients, health insurances, and welfare systems [154]. Cost-effectiveness considerations should guide cost definition [155,156]. In this context, the availability of competitive drugs from one side, and the promotion of repurposing old, inexpensive drugs from the other side, may facilitate wider access to effective therapies.

## Figures and Tables

**Table 1:** Oral drugs for the treatment of ATTR amyloidosis.

Drug [References]	Absorption	Volume of Distribution	Protein Binding	Metabolism	Route of Elimination	Half-life	Clearance	Dosing *
Tafamidis and tafamidis meglumine [68,69,70]	Peak plasma concentration within 4 h following oral administration	18.5 L	99.9% protein bound in plasma, mostly to transthyretin	Largely not subject to first pass or oxidative metabolism, being 90% unchanged after in vitro experiments. Mainly metabolized through glucuronidation and excreted in bile.	20 mg oral dose: 59% recovered in the feces, largely as unchanged drug; 22% recovered in the urine, mostly as the glucuronide metabolite	49 h	Oral clearance: 0.263 L/h. Apparent total clearance: 0.44 L/h.	20 mg or 80 mg (61 mg of tafamidis) QD
Diflunisal [71,72,73,74,75,76,77,78]	Bioavailability of 80–90%. Peak plasma concentration within 2–3 h following oral administration	Not available	98 to 99% protein bound in plasma	Hepatic metabolism, primarily via glucuronide conjugation (90% of the administered dose).	Excreted in the urine as two soluble glucuronide conjugates accounting for about 90% of the administered dose. Little or no excretion in the feces.	8–12 h	Not available.	250 mg BID
Acoramidis [79,80,81,82,83,84,85]	Peak plasma concentration within 1 h following oral administration	Not available	High binding selectivity for TTR. No data on binding to albumin or plasma proteins other than TTR	Predominantly acyl glucuronidation based on in vitro studies.	Up to 9.5% excreted as intact AG10 in urine. 19.5–23.5% extreted as AG10 acylglucuronide. No data on fecal elimination.	25 h	1.58–5.98 L/h.	800 mg BID
Tolcapone [86,87,88]	Absolute bioavailability of about 65%	9 L	>99.9% (to serum albumin)	Mainly metabolized through glucuronidation.	Almost completely metabolized before excretion, with only a very small amount (0.5% of dose) found unchanged in the urine. The glucuronide conjugate is mainly excreted in the urine but is also excreted in the bile.	2–3.5 h	7 L/h	NA
Doxycycline [89,90,91,92,93]	Peak plasma concentration within 2 hours following oral administration	0.7 L/kg	>90%	Metabolized in the liver and gastrointestinal tract and concentrated in bile. Major metabolic pathways have not been identified.	Mainly the urine-(40–60%) and feces (30%) as active and unchanged drug.	18–22 h	Excretion by the kidney is about 40% over 72 h in individuals with normal kidney function.	100 mg BID

* Dosing refers to more common dosing in the context of treatment for ATTR amyloidosis; Pharmacokinetics data are derived from each drug’s label or from [79] for acoramidis. QD, quaque die; BID, bis in die; NA, not available.

## Data Availability

Not applicable.
